# Evaluation of Central Corneal Thickness Using Corneal Dynamic Scheimpflug Analyzer Corvis ST and Comparison with Pentacam Rotating Scheimpflug System and Ultrasound Pachymetry in Normal Eyes

**DOI:** 10.1155/2015/767012

**Published:** 2015-12-01

**Authors:** Ayong Yu, Weiqi Zhao, Giacomo Savini, Zixu Huang, Fangjun Bao, Weicong Lu, Qinmei Wang, Jinhai Huang

**Affiliations:** ^1^School of Ophthalmology and Optometry, Wenzhou Medical University, 270 West Xueyuan Road, Wenzhou, Zhejiang 325027, China; ^2^Key Laboratory of Vision Science, Ministry of Health of the People's Republic of China, Wenzhou, Zhejiang, China; ^3^G. B. Bietti Foundation IRCCS, Rome, Italy

## Abstract

*Purpose.* To assess the repeatability and reproducibility of central corneal thickness (CCT) measurements by corneal dynamic Scheimpflug analyzer Corvis ST in normal eyes and compare the agreement with Pentacam rotating Scheimpflug System and ultrasound pachymetry.* Methods.* 84 right eyes underwent Corvis ST measurements performed by two operators. The test-retest repeatability (TRT), within-subject coefficient of variation (CoV), and intraclass correlation coefficient (ICC) were used to evaluate the intraoperator repeatability and interoperator reproducibility. CCT measurements also were obtained from Pentacam and ultrasound pachymetry by the first operator. The agreement between the three devices was evaluated with 95% limits of agreement (LoA) and Bland-Altman plots.* Results.* Corvis ST showed high repeatability as indicated by TRT ≤ 13.0 *μ*m, CoV < 0.9%, and ICC > 0.97. The interoperator reproducibility was also excellent. The CoV was <0.9%, and ICC was >0.97. Corvis ST showed significantly lower values than Pentacam and ultrasound pachymetry (*P* < 0.001). The 95% LoA between Corvis ST and Pentacam or ultrasound pachymetry were −15.8 to 9.5 *μ*m and −27.9 to 12.3 *μ*m, respectively.* Conclusions.* Corvis ST showed excellent repeatability and interoperator reproducibility of CCT measurements in normal eyes. Corvis ST is interchangeable with Pentacam but not with ultrasound pachymetry.

## 1. Introduction

Accurate assessment of the central corneal thickness (CCT) has become extremely important in ophthalmologic examinations. Preoperatively, it helps the ophthalmologist to safely plan corneal refractive procedures and screen for refractive surgery candidates, in order to reduce the risk of postoperative complications [1]. Besides, CCT measurements play a crucial role in the diagnosis and management of glaucoma because the value of intraocular pressure should be adjusted in accordance with CCT [2]. CCT measurements also play an important role in the diagnosis of corneal diseases, such as Fuchs' corneal dystrophy and keratoconus [3, 4].

For many years, ultrasound pachymetry has been the most frequently used method to measure CCT because it is relatively inexpensive and easy to use and has high intraoperator repeatability [2, 5]. Nevertheless, ultrasound pachymetry has certain disadvantages, such as corneal-probe contact, the need for topical anesthesia, and the risk for transmission of infections and corneal epithelial lesions [6]. Besides, the reliability of ultrasound pachymetry results depends on the operator's skill when placing the probe perpendicularly to the cornea. Over the last decade, many noncontact devices have been developed. Among these, Scheimpflug technology plays a major role, including Pentacam (Oculus, Wetzlar, Germany), Sirius (Costruzione Strumenti Oftalmici, Florence, Italy), Galilei (Ziemer, Port, Switzerland), and TMS-5 (Tomey, Nagoya, Japan). Previous studies have shown that common used device Pentacam has high intraoperator repeatability and interoperator reproducibility for CCT measurements [7–9].

The corneal dynamic Scheimpflug analyzer Corvis ST (Oculus Optikgeräte, Inc., Wetzlar, Germany) is relatively new, noncontact corneal biomechanics equipment, which is composed of an air puff indentation system and ultra-high-speed Scheimpflug technology. The ultra-high-speed Scheimpflug camera has a blue light LED and acquires the deformation process at 4330 frames/s with an 8 mm horizontal coverage. Because of the air impulse, the cornea experiences three stages: first applanation, highest concavity, and second applanation. Furthermore, CCT is obtained by the corneal initial state of the central horizontal cross-section diagram through the Scheimpflug technology. Few studies [10–12] have evaluated the intraoperator repeatability of CCT measurements obtained by this device in normal population. Ali et al. [10] evaluated the intersession reproducibility of the CCT at different times of the day with this device. Chen et al. [13] assessed the intraoperator repeatability and interobserver reproducibility in virgin and post-PRK eyes but only applied single value to evaluate interobserver reproducibility. However, to our knowledge, the interoperator reproducibility of Corvis ST for CCT measurements using both single and average measurement value methods in normal eyes has not yet been evaluated.

The purpose of the present study was to prospectively assess the intraoperator repeatability and interoperator reproducibility of CCT measurements using both single and average methods acquired from the Corvis ST in normal eyes and compare the agreement with Pentacam and ultrasound pachymetry.

## 2. Subjects and Methods

This prospective study was conducted on normal subjects recruited from the staff and students of the Eye Hospital of Wenzhou Medical University. The research protocol conformed to the tenets of the Declaration of Helsinki and was approved by the Office of Research Ethics, Eye Hospital of Wenzhou Medical University. Informed consent was acquired from all subjects after explaining the purpose of the study.

The exclusion criteria were active ocular pathology, any history of ocular surgery or trauma, recent contact lens wear (soft contact lens within two weeks and rigid contact lens within four weeks), systemic diseases with eye symptoms, and intraocular pressure >21 mmHg.

All subjects underwent a comprehensive ophthalmic examination, including uncorrected distance visual acuity, best-corrected visual acuity, slit-lamp microscopy, noncontact tonometer, and fundus examinations. Subsequently, we applied Pentacam, Corvis ST, and ultrasound pachymetry to measure CCT. To avoid any effect of the ultrasound probe and topical anesthetic on the cornea, the two noncontact pieces of equipment were used first. The sequence of measurements with Pentacam and Corvis ST was randomly chosen. Measurements were acquired from the right eyes of subjects to avoid structural similarities between fellow eyes [14]. In order to minimize the diurnal variation on CCT readings, all measurements were performed from 10:00 to 17:00. The subjects were required to completely blink twice before measurements, in order to form an optically smooth tear film on the cornea.

Corvis ST examination applies four red alignment marks to position the center of the cornea on the computer screen. Once positioned successfully, a puff of air with a pressure of 25 kPa is emitted automatically from the instrument aimed at the cornea at a distance of 11 mm. During the examination, the Scheimpflug camera records corneal deformation process and CCT. The process was repeated until three acceptable readings were obtained. The three CCT measurements obtained by each examiner were used to evaluate the intraoperator repeatability. The mean value of three successive measurements and the first measurement by each examiner were used to analyze the interoperator reproducibility.

The Pentacam was used as previously described [8, 9]. Briefly, the subject was instructed to sit, open both eyes, and fixate on a target within the device. The real-time image of the subject's eye on the computer screen was adjusted according to the pupil edge, center, and the corneal apex by moving the joystick. To avoid operator-dependent variables, the automatic release mode was applied. The Pentacam would automatically measure when correct alignment with the corneal apex and focus was achieved. Only when the “examination quality specification” reading showed OK, it was recorded; otherwise it was excluded and remeasured until three valid readings were obtained.

After the CCT measurements were obtained by Pentacam and Corvis ST, an A-scan ultrasound pachymetry (SP-3000, Tomey Inc., Nagoya, Japan) was used. Before the measurements, the instrument was calibrated by an experienced technician. First, the cornea was anesthetized with topical 0.5% proparacaine hydrochloride (Alcaine; Alcon Laboratories, TX, USA). Then, the subject in the supine position was asked to fixate on a target on the ceiling. The examiner placed the pachymeter probe on the central cornea as perpendicularly as possible. Then, five consecutive measurements were obtained, of which the highest and lowest were excluded, and the remaining three were recorded.

*Statistical Analysis*. Statistical analysis was performed using SPSS software for Windows version 21 (IBM Corporation, USA) and Microsoft Office Excel (Microsoft Corp., WA, USA). *P* < 0.05 was considered to be statistically significant. The distributions were checked by Kolmogorov-Smirnov test, which showed that the data were normally distributed (*P* > 0.05). Results were presented as means ± standard deviations.

To assess the intraoperator repeatability of Corvis ST, within-subject standard deviation (*S*_*w*_), test-retest repeatability (TRT), within-subject coefficient of variation (CoV), and intraclass correlation coefficient (ICC) were calculated for the three successive measurements obtained by the two operators. The TRT is 2.77 times *S*_*w*_, which represents an interval within which 95% of the differences between measurements are expected to lie [15]. The CoV is defined as the ratio of *S*_*w*_ to the overall mean. A lower CoV is closely related to higher repeatability. The ICC represents the consistency of measurement. The closer the ICC is to 1, the better the consistency of measurement is. To evaluate the interoperator reproducibility of Corvis ST, the average method (the difference between the mean of the three successive measurements obtained by the two operators) and the single method (the first measurement of each operator) were used. Then, the interoperator *S*_*w*_, TRT, CoV, and ICC were also calculated.

For multiple comparisons between CCT measurements obtained by Corvis ST and Pentacam or ultrasound pachymetry, the repeated-measures analysis of variance (ANOVA) with Bonferroni post hoc comparison test was used. Furthermore, the 95% limits of agreement (LoA) were calculated and Bland-Altman plots were produced to evaluate the agreement on the CCT measurements between Corvis ST versus Pentacam and Corvis ST versus ultrasound pachymetry [16]. The 95% LoA are defined as mean ± 1.96 SD, which represent an interval within which 95% of the differences between readings are expected to lie [8].

## 3. Results

This study enrolled 84 right eyes of 84 subjects (38 males and 46 females). The mean age of the subjects was 27.30 ± 6.06 years (range 18 to 49 years). The mean spherical equivalent refraction was −4.12 ± 2.66 D (range −10.50 to +0.50 D).

### 3.1. Intraoperator Repeatability

The CCT measurements obtained using Corvis ST showed excellent intraoperator repeatability for both operators (Table 1). The TRT values were ≤13 *μ*m, the CoV values were <0.9%, and the ICC values were >0.97.

### 3.2. Interoperator Reproducibility

The mean ± SD of CCT, *S*_*w*_, TRT, CoV, and ICC of Corvis ST are shown in Table 2, which demonstrate high interoperator reproducibility. In the average method, the TRT was 9.7 *μ*m, the CoV was 0.65%, and the ICC was 0.98. In the single method, the TRT was 12.6 *μ*m, the CoV was 0.85%, and the ICC was 0.97. Obviously, the error of the average method was smaller than the single method.

### 3.3. Agreement between Corvis ST, Pentacam, and Ultrasound Pachymetry

**Figure 1:**
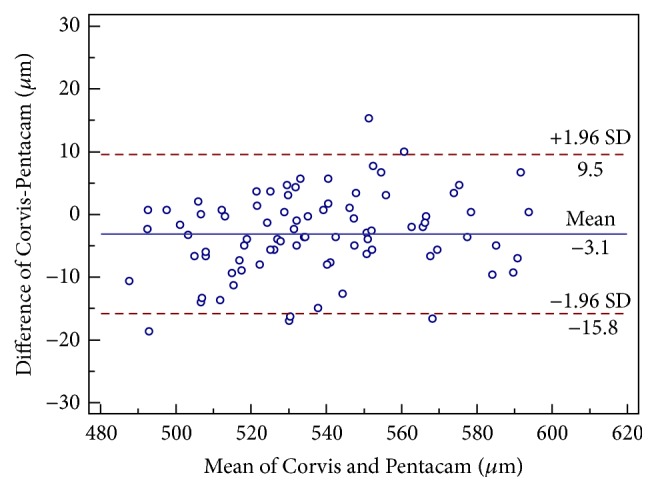
Bland-Altman plot demonstrating central corneal thickness measurements obtained using corneal dynamic Scheimpflug analyzer Corvis ST and Pentacam rotating Scheimpflug system against the mean values for both devices. The 95% limits of agreement are represented as the upper and lower lines.

**Figure 2:**
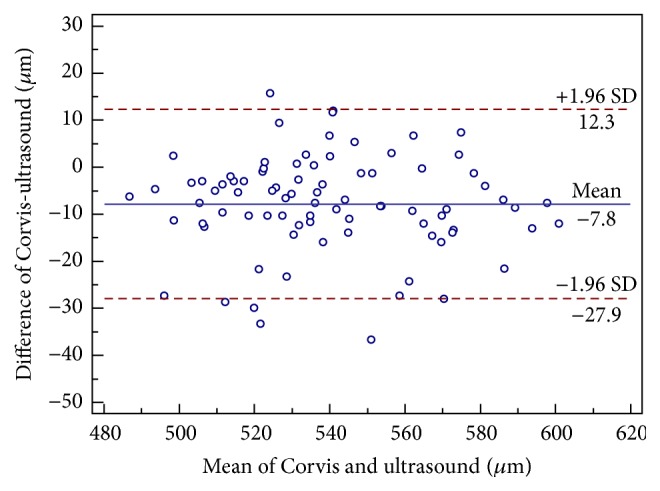
Bland-Altman plot demonstrating central corneal thickness measurements obtained using corneal dynamic Scheimpflug analyzer Corvis ST and ultrasound pachymetry against the mean values for both devices. The 95% limits of agreement are represented as the upper and lower lines.

The mean CCT readings using Corvis ST, Pentacam, and ultrasound pachymetry were 535.9 ± 27.0 *μ*m, 539.0 ± 25.70 *μ*m, and 543.7 ± 27.52 *μ*m, respectively. The CCT readings measured by Corvis ST were significantly thinner than Pentacam (*P* < 0.001). Bland-Altman analysis confirmed these results (Table 3 and Figure 1). The mean difference was −3.2 *μ*m (95% LoA, −15.8 to 9.5 *μ*m). CCT measurements between Corvis ST and ultrasound pachymetry were significantly different (*P* < 0.001). The mean difference was −7.8 *μ*m (95% LoA, −27.9 to 12.3 *μ*m) (Table 3 and Figure 2).

## 4. Discussion

The present study was prospectively designed to assess (1) the intraoperator repeatability and (2) the interoperator reproducibility by applying the single and average methods on CCT measurements obtained from Corvis ST in normal eyes and (3) to evaluate the agreement between Corvis ST, Pentacam, and ultrasound pachymetry. The TRT was ≤13.0 *μ*m, the CoV was <0.90%, and the ICC was >0.97, which represented high intraoperator repeatability in CCT readings using Corvis ST in normal eyes. Ali et al. [10] obtained similar results in normal eyes, with TRT, CoV, and ICC of 27 *μ*m, 1.83%, and 0.95, respectively. Hon and Lam [11] reported TRT, CoV, and ICC of 15.34 *μ*m, 1.01%, and 0.96, respectively, in normal subjects. Nemeth et al. [12] obtained similar ICC of 0.97 and CoV of 0.8% for CCT in normal eyes. Salvetat et al. [17] reported ICC of 0.99 in normal subjects and primary open-angle glaucoma patients. Chen et al. [13] obtained similar results in virgin eyes, with TRT, CoV, and ICC of 12.56 *μ*m, 0.69%, and 0.99, respectively. However, they reported worse results in post-PRK eyes, with TRT and CoV of 22.61 *μ*m and 2.29%, respectively.

Previous studies had assessed the intraoperator repeatability of CCT values using other Scheimpflug systems and specular microscopes, such as Pentacam, Sirius, Galilei, Orbscan II (Bausch & Lomb, Rochester, NY, USA), and SP-02 (Costruzione Strumenti Oftalmici, Italy). Huang et al. [7] evaluated the intraoperator repeatability of Sirius and Pentacam, a rotating Scheimpflug camera combined with a Placido disk corneal topographer and a rotating Scheimpflug camera, respectively, in normal subjects. They indicated that the TRT values were 8.79 *μ*m and 9.65 *μ*m, the CoV values were 0.59% and 0.65%, and the ICC values were 0.98 and 0.98, respectively. These results were slightly better than our results with Corvis ST. Al-Mohtaseb et al. [18] assessed the intraoperator repeatability of the Galilei, a dual rotating Scheimpflug camera combined with a Placido disk, in normal eyes. The CoV was 0.36% and the ICC was 0.99, which were also slightly better than our results with Corvis ST. Maldonado et al. [19] studied the intraoperator repeatability of the Orbscan II, a scanning-slit combined with Placido disc topography, with TRT and CoV of 20.2 *μ*m and 1.5%, respectively, which were worse than our results. Bao et al. [20] assessed the intraoperator repeatability of SP-02, a noncontact specular microscope, in normal eyes with TRT, CoV, and ICC of 18.67 *μ*m, 1.23%, and 0.97, respectively, which were worse than our results with Corvis ST. These indirect comparisons indicate that the above-mentioned devices have high intraoperator repeatability, with Galilei being the best. The Galilei has two opposite Scheimpflug cameras and can calculate the average value of the CCT obtained by the two cameras. This reduces the artifact error caused by ocular movements and increases repeatability [21]. Therefore, future studies should compare Corvis ST, Galilei, and Sirius.

In the present study, we analyzed the interoperator reproducibility of CCT measurements acquired with Corvis ST by applying the single and average methods. The TRT and CoV of the mean values were smaller than the single values. Chen and Lam [22, 23] demonstrated that the width of 95% LoA was reduced by using an averaged result rather than the first result of each visit. Ali et al. [10] assessed the reproducibility of the CCT with the device in normal eyes, which was intersession reproducibility at different times of the day. In their study, TRT, CoV, and ICC were 11.0 *μ*m, 7.41%, and 0.995, respectively, while they were 9.70 *μ*m, 0.65%, and 0.984 in the current study, respectively. Our results were obviously better than their results because our results were accomplished within 30 minutes, which eliminated the effects of different times on the CCT. Chen et al. [13] measured the interoperator reproducibility of the CCT by the single method in virgin and post-PRK eyes, and the TRT, CoV, and ICC were 13.24 *μ*m, 0.72%, and 0.98 in virgin eyes and 9.89 *μ*m, 0.58%, and 1.00 in post-PRK eyes, respectively, which were similar to our results using the single method. Salvetat et al. [17] only applied ICC to assess the interoperator reproducibility, which was 0.99 in normal subjects and primary open-angle glaucoma patients. We believe that ours is the first study to evaluate the interoperator reproducibility of Corvis ST using the average and single methods with TRT, CoV, and ICC.

In addition, we compared the CCT readings between Corvis ST, Pentacam, and ultrasound pachymetry in normal eyes. Corvis ST had a slightly lower CCT measurement as compared to Pentacam with a mean of 3.2 *μ*m. Meanwhile, Corvis ST significantly underestimated CCT as compared to ultrasound pachymetry with an average of 7.8 *μ*m. The 95% LoA between Corvis ST and Pentacam were narrow and comparable, with the CCT diurnal pachymetric variation range of −11 to 11 *μ*m [24]. Therefore, Corvis ST and Pentacam could be interchangeably used in normal eyes in most clinical applications. However, Corvis ST cannot be interchangeably used with ultrasound pachymetry in normal eyes because of broad 95% LoA between the two devices. Our results were similar to or better than those previously reported when investigating agreement of CCT measurements obtained from other Scheimpflug systems, Orbscan II, and specular microscopes with respect to Pentacam or ultrasound pachymetry (Table 4). Several reasons may explain the difference in CCT readings between Corvis ST and ultrasound pachymetry. Firstly, topical 0.5% proparacaine hydrochloride may cause corneal thickness to increase by 8.6 *μ*m in 80 seconds [25]. Secondly, the accuracy of ultrasound pachymetry depends on the operator's proficiency and whether the corneal probe is perpendicularly placed on the center of the cornea. Thirdly, if the posterior surface reflection point is closer to the anterior chamber, the CCT measurement is thicker than the actual value [26].

The present study had some limitations. First, we only assessed the intraoperator repeatability and interoperator reproducibility in normal subjects and did not include keratoconus, glaucoma, or postrefractive surgery patients. Further research is needed to assess the intraoperator repeatability and interoperator reproducibility in the above-mentioned patients. Second, our study is restricted by the different algorithms each device uses for obtaining the CCT. The CCT obtained by Corvis ST and Pentacam are derived from the corneal apex. However, ultrasound pachymetry is performed over the pupil center, and its position depends on the operator's experience.

In conclusion, Corvis ST showed high intraoperator repeatability and interoperator reproducibility of CCT measurements in normal eyes. Corvis ST and Pentacam showed excellent agreement, which suggests that the two devices may be interchangeably used for CCT measurements in the clinical setting. However, the CCT readings between Corvis ST and ultrasound pachymetry are not directly interchangeable owing to the relatively wide 95% LoA.

## Figures and Tables

**Table 1:** Intraobserver repeatability of the corneal dynamic Scheimpflug analyzer Corvis ST in measuring central corneal thickness (*N* = 84).

Operator	Mean (*μ*m) ± SD	*S*_*w*_ (*μ*m)	TRT (*μ*m)	CoV (%)	ICC (95% CI)
1st	535.9 ± 27.0	4.8	13.0	0.87	0.971 (0.958 to 0.980)
2nd	537.4 ± 27.6	4.7	13.0	0.87	0.972 (0.960 to 0.981)

SD = standard deviation, *S*_*w*_ = within-subject standard deviation, TRT = test-retest repeatability (2.77*S*_*w*_), CoV = within-subject coefficient of variation, and ICC = intraclass correlation coefficient.

**Table 2:** Interobserver reproducibility of central corneal thickness readings using average (from average of 3 consecutive readings from each observer) and single (from the first reading from each observer) measurement by the corneal dynamic Scheimpflug analyzer Corvis ST.

Parameter	Mean (*μ*m) ± SD	*S*_*w*_ (*μ*m)	TRT (*μ*m)	CoV (%)	ICC (95% CI)
Average	536.6 ± 27.2	3.5	9.7	0.65	0.984 (0.973 to 0.990)
Single	536.4 ± 27.5	4.5	12.6	0.85	0.973 (0.958 to 0.982)

SD = standard deviation, *S*_*w*_ = within-subject standard deviation, TRT = test-retest repeatability (2.77*S*_*w*_), CoV = within-subject coefficient of variation, and ICC = intraclass correlation coefficient.

**Table 3:** Comparison of the central corneal thickness readings obtained using the corneal dynamic Scheimpflug analyzer Corvis ST, Pentacam rotating Scheimpflug system, and ultrasound pachymetry.

Device pairings	Mean difference (*μ*m) ± SD	95% LoA (*μ*m)	*P* value
Corvis-Pentacam	−3.2 ± 6.5	−15.8 to 9.5	<0.001
Corvis-USP	−7.8 ± 10.3	−27.9 to 12.3	<0.001

USP = ultrasound pachymetry, SD = standard deviation.

**Table 4:** Summary of previous studies for the agreement of CCT measurements obtained by other Scheimpflug systems, Orbscan II and specular microscopes in comparison to Pentacam or ultrasound pachymetry.

Author (year)	Patients/eyes	Device pairings	Mean difference (*μ*m) ± SD	*P* value	95% LoA (*μ*m)
Lanza et al. [27] (2015)	102/102	Pentacam-Orbscan II	13.66 ± 16.53	<0.0001	−18.74 to 46.06
		Sirius-Orbscan II	15.18 ± 17.16	<0.0001	−18.45 to 48.81
		Sirius-Pentacam	1.52 ± 6.21	0.015	−10.65 to 13.69

Khaja et al. [28] (2015)	32/32	USP-Orbscan II	2.8 ± 0.28	NA	−30.15 to 24.40
		USP-specular microscopy	8.69 ± 1.24	NA	−8.82 to 27.4

Smedowski et al. [29] (2014)	76/152	Corvis ST-Pentacam	NA	>0.05	NA
		Corvis ST-USP	NA	>0.05	NA

Huang et al. [30] (2014)	66/66	Pentacam-Sirius	−3.3 ± 5.2	<0.001	−13.6 to 6.9
		Pentacam-Galilei	−9.3 ± 3.7	<0.001	−16.6 to −2.0
		Sirius-Galilei	−6.0 ± 4.0	<0.001	−13.8 to 1.9

Anayol et al. [31] (2014)	32/32	Galilei-Pentacam	13.93 ± 0.88	<0.001	11.74 to 16.12
		Galilei-Sirius	14.66 ± 0.69	<0.001	12.96 to 16.37
		Pentacam-Sirius	0.73 ± 0.93	1.0	−1.50 to 3.02

Maresca et al. [32] (2014)	35/35	Sirius-USP	−13.9 ± 14.4	<0.001	−42.2 to 14.4

Feizi et al. [33] (2014)	88/88	USP-Orbscan	−14.5 ± 22.9	<0.001	−59.4 to 30.4
		USP-Galilei	−16.0 ± 19.6	<0.001	−54.5 to 22.5
		Orbscan-Galilei	−1.5 ± 17.0	0.99	−34.8 to 31.9

De La Parra-Colín et al. [34] (2014)	16/16	Sirius-Pentacam	−10.1 ± 9.0	NA	−27.7 to 7.5

Jorgel et al. [35] (2013)	50/50	Sirius-USP	4.68 ± 10.47	0.003	−15.84 to 25.20

Bayhan et al. [36] (2014)	50/50	USP-Sirius	17.58 ± 8.13	<0.001	15.27 to 19.89

Huang et al. [37] (2013)	43/43	Sirius-USP	6.88 ± 6.77	0.000	−6.39 to 20.14

Nassiri et al. [38] (2014)	32/61	Pentacam-USP	−1 ± 9	0.32	−20 to 17
		Orbscan II-USP	6 ± 14	<0.001	−21 to 33

Al Farhan et al. [39] (2013)	30/30	USP-specular microscopy	−2.40 ± 9.10	0.16	−38.70 to 39.90

Tai et al. [40] (2013)	92/184	Pentacam-USP	10.08 ± 10.96	0.012	−11.40 to 31.56
		Specular microscopy-USP	−20.49 ± 8.91	<0.001	−37.95 to −3.04
		Specular microscopy-Pentacam	−30.57 ± 10.26	<0.001	−59.69 to −10.45

Chen et al. [41] (2012)	35/35	Pentacam-USP	5.27 ± 9.55	0.007	−24.0 to −13.4

Aramberri et al. [42] (2012)	35/35	Pentacam-Galilei	−2.76 ± 4.52	<0.01	−6.1 to 11.6

González-Pérez et al. [43] (2011)	22/22	USP-Pentacam	3 ± 10	0.233	−16.2 to 21.2
		USP-Orbscan II	32 ± 15	<0.001	3.1 to 60.8
		USP-specular microscopy	26 ± 37	0.004	−46.2 to 97.8
		Pentacam-Orbscan II	29 ± 11	<0.001	7.2 to 51.6
		Pentacam-specular microscopy	23 ± 32	0.003	−40.2 to 86.2
		Orbscan II-specular microscopy	−6 ± 35	0.399	−74.8 to 62.0
		USP-Orbscan II	−15 ± 17	0.001	−47.9 to 18.7
		Pentacam-Orbscan II	−17 ± 14	<0.001	−43.9 to 9.8
